# Picosecond sulfur K-edge X-ray absorption spectroscopy with applications to excited state proton transfer

**DOI:** 10.1063/1.4983157

**Published:** 2017-05-08

**Authors:** Benjamin E. Van Kuiken, Matthew R. Ross, Matthew L. Strader, Amy A. Cordones, Hana Cho, Jae Hyuk Lee, Robert W. Schoenlein, Munira Khalil

**Affiliations:** 1 Department of Chemistry, University of Washington, Seattle, Washington 98195, USA; 2 Ultrafast X-ray Science Laboratory, Chemical Sciences Division, Lawrence Berkeley National Laboratory, Berkeley, California 94720, USA

## Abstract

Picosecond X-ray absorption (XA) spectroscopy at the S K-edge (∼2.4 keV) is demonstrated and used to monitor excited state dynamics in a small organosulfur molecule (2-Thiopyridone, 2TP) following optical excitation. Multiple studies have reported that the thione (2TP) is converted into the thiol (2-Mercaptopyridine, 2MP) following photoexcitation. However, the timescale and photochemical pathway of this reaction remain uncertain. In this work, time-resolved XA spectroscopy at the S K-edge is used to monitor the formation and decay of two transient species following 400 nm excitation of 2TP dissolved in acetonitrile. The first transient species forms within the instrument response time (70 ps) and decays within 6 ns. The second transient species forms on a timescale of ∼400 ps and decays on a 15 ns timescale. Time-dependent density functional theory is used to identify the first and second transient species as the lowest-lying triplet states of 2TP and 2MP, respectively. This study demonstrates transient S K-edge XA spectroscopy as a sensitive and viable probe of time-evolving charge dynamics near sulfur sites in small molecules with future applications towards studying complex biological and material systems.

## INTRODUCTION

In recent years, picosecond and femtosecond time-resolved X-ray spectroscopies have become sensitive probes of time-evolving molecular structural dynamics in photoexcited materials and molecular systems in the condensed phase.^1,2^ X-ray absorption (XA) spectroscopy is unique in its ability to measure dynamics in solution with atomic specificity by probing excitations of highly localized core electrons to unoccupied valence orbitals. This is unlike more common transient spectroscopies in the optical and IR regimes, which often report on delocalized electronic and vibrational states. Synchrotron and X-ray free electron laser (XFEL) facilities are being used to perform transient metal K and L-edge XA and X-ray emission measurements. These experiments have proven to be effective probes of metal-ligand bonding interactions, charge and electron transfer dynamics, and spin crossover phenomena in solution.^3–17^ Ultrafast XA and emission spectroscopies at the O and N K-edges have been used to study hydrogen bonding dynamics, proton transfer, and spin crossover phenomena.^18–21^ Recently, S K-edge spectroscopy at a specific time-delay has been applied to identify photoproducts following the photoexcitation of an aromatic thiol.^22^ In this work, we extend time-resolved S K-edge spectroscopy to identify the formation and follow the dynamics of multiple photoproducts during an excited state proton transfer (ESPT) reaction in the solution.

The transfer of protons between bonding sites is pivotal to many biological and chemical systems. The basic mechanistic details by which photoinduced proton transfer takes place are often studied by time-resolved spectroscopy in the optical and IR regimes.^23–26^ Proton transfer events involving sulfur atoms are common both in biological systems and in organic photochemistry where thiol groups act as proton donors. S K-edge XA spectroscopy has been widely used to study metal-ligand interactions in bioinorganic chemistry, but it is also a sensitive probe of speciation in organosulfur molecules.^27–30^ The S K-edge is sensitive to both the charge on the S atom and the character of the low-lying valence orbitals due to the dipole allowed 1s → nπ transitions, making it an ideal probe of proton transfer events taking place at or near the S atom.

**FIG. 1.**
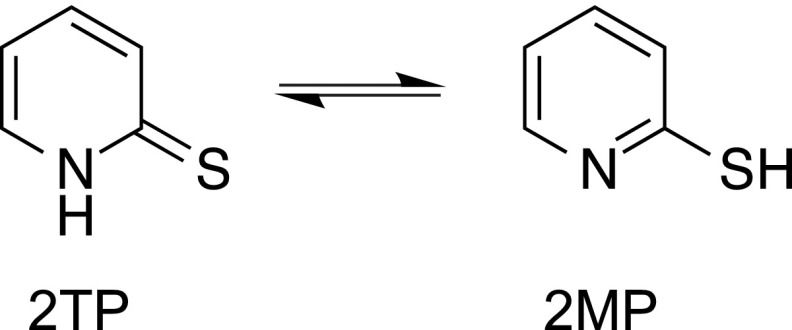
The tautomeric equilibrium of 2-thiopyridone (2TP) and 2-mercaptopyridine (2MP).

In this work, we use S K-edge XA spectroscopy to probe the structural dynamics in 2-Thiopyridone (2TP, C_5_H_5_NS) following 400 nm excitation. 2TP has many uses, ranging from general chemical synthesis, antimicrobial, and antifungal uses^31,32^ to polymer and gold surface linkers for the rapid purification of DNA^33^ and surface enhanced Raman spectroscopy.^34^ 2TP exists in equilibrium with its tautomer, 2-mercaptopyridine (2MP), as depicted in Figure 1. The ground state energy differences of the two 2TP and 2MP forms differ by 10–15 kJ/mol depending on the solvent.^35–40^ The 2MP isomer is known to be dominant in vapor, whereas the 2TP form dominates in polar solvents. In non-polar solvents, there exists an equilibrium between the two isomers and the solid is known to be a 2TP dimer.^41,42^ Several studies have reported that 2TP undergoes ESPT forming the thiol 2MP.^38,43^ Based on nanosecond time-resolved Raman measurements, Du *et al*. suggested that 2MP in water is formed in its singlet ground state within tens of nanoseconds, but they were not able to identify intermediate states, leading to 2MP formation.^38^ Similarly, the flash photolysis studies of Alam *et al*. identified the formation of 2MP in acetonitrile.^43^ This study suggested that 2MP is formed following the initial population of the 2TP triplet state, but the authors were not able to draw conclusions regarding the electronic state of the 2MP product.

We exploit the sensitivity of the S K-edge to follow the photochemical reaction pathways of 2TP dissolved in acetonitrile following 400 nm excitation. The 400 nm excitation pulse is resonant with the S_2_ state of 2TP (S_1_ is a dark state), and it is conceivable that relaxation from the S_2_ state could result in the formation of the T_1_ state of 2TP or either the T_1_ or S_0_ state of 2MP. Transient S K-edge spectra indicate the formation and decay of multiple photoproducts on the ps-ns timescales. Time-dependent density functional theory (TDDFT) is used to calculate S K-edge XA spectra of possible transient photochemical species and allows us to identify a photoexcited triplet state formed within the instrument response time and an ESPT product forming on a 400 ps timescale. To the best of our knowledge, this work is among the first to follow the excited state structural dynamics of multiple photoproducts by time-resolved S-K-edge XA spectroscopy.

## EXPERIMENTAL AND COMPUTATIONAL METHODS

Experiments on the S K-edge of 2TP were performed at BL 6.0.1 at the Advanced Light Source (ALS), and the details of the experimental setup have been given in multiple papers.^13,44^ A two-crystal Si (111) monochromator selects a narrow spectral region of the incident X-rays (resolving power, ΔE/E ∼ 1000), which is then focused to about 200 *μ*m at the sample. X-ray transmission is detected on an avalanche diode, which is amplified, gated using a boxcar integrator, and then recorded using a computer. For time resolved XA spectroscopy, 400 nm laser pump pulses are used for photo-excitation. Laser pulses are produced from an amplified Ti:Sapphire laser system described elsewhere.^45^ The 70 fs 800 nm pulses from the laser are loosely focused into a β-barium borate (BBO) crystal to generate 400 nm light. To avoid excessive peak powers, which may disrupt liquid flow, the 400 nm pulse is temporally stretched with a fused silica block to lengthen the pulse to approximately 2 ps. The laser beam is routed to the X-ray chamber, aligned near-collinear to the X-ray path, and focused to a 250 *μ*m spot size at the sample position with an energy of 0.16 mJ/pulse. The transient XA experiments were performed in the transmission mode with a 0.120 M solution of 2TP in acetonitrile. All materials were purchased from Sigma Aldrich and used without further purification. A flowing liquid jet of 50 *μ*m was used in the experiment. Experiments were performed in a vacuumed chamber backfilled with 1 atm of helium, which is required for maintaining high X-ray flux and providing sufficient pressure for stable liquid flow. The X-ray and laser beams were overlapped at their foci using pinholes and knife-edges to ensure the matched location and size, respectively. The laser is synchronized to the storage ring and runs at a repetition rate of 2 kHz. The X-ray beam is measured at 4 kHz to obtain X-ray transmission intensities of $I_{on}$ and $I_{off}$ for laser on and off, respectively. We then calculate the differential absorption spectrum as $\Delta A=-{\text{log}}_{10}\left(1+\frac{I_{on}-I_{off}}{I_{off}}\right)$. Transient XA spectra were obtained by fixing the pump-probe delay time and scanning the X-ray monochromator. The time-evolution of certain spectral features was obtained by fixing the monochromator and scanning delay times. In either case, 30–90 s of averaging was recorded for each data point in a given measurement. Equilibrium XA spectra were collected in the total fluorescence yield mode with a silicon photodiode. X-ray energies were calibrated with 50 mM sodium thiosulfate, where the white line feature is known to occur at 2470.02 eV.^46^

All TDDFT calculations were performed using the ORCA quantum chemistry program package.^47^ The geometries were separately optimized for 2MP, 2TP, and the associated triplet states of the isomers. All calculations employed the B3LYP functional and the def2-TZVP(-f) basis set, which is identical to the parent basis set except for the removal of f functions from first row atoms.^48,49^ Calculations employed the COSMO model to mimic the effects of the acetonitrile solvent (ε = 36.6; *n* = 1.344).^50^ Scalar relativistic effects were accounted for by using the ZORA model.^51,52^ All TDDFT calculations were performed within the Tamm-Dancoff approximation to the full linear-response formalism, and core-excitations were obtained by only including the S 1s orbital in the donor space. Due to deficiencies in functionals and basis sets, calculated X-ray spectra are typically energetically shifted with respect to experimental data, and it is conventional to apply a uniform shift prior to comparison with the experiment. A shift of +40.9 eV was applied to all spectra presented below.

## RESULTS AND DISCUSSION

**FIG. 2.**
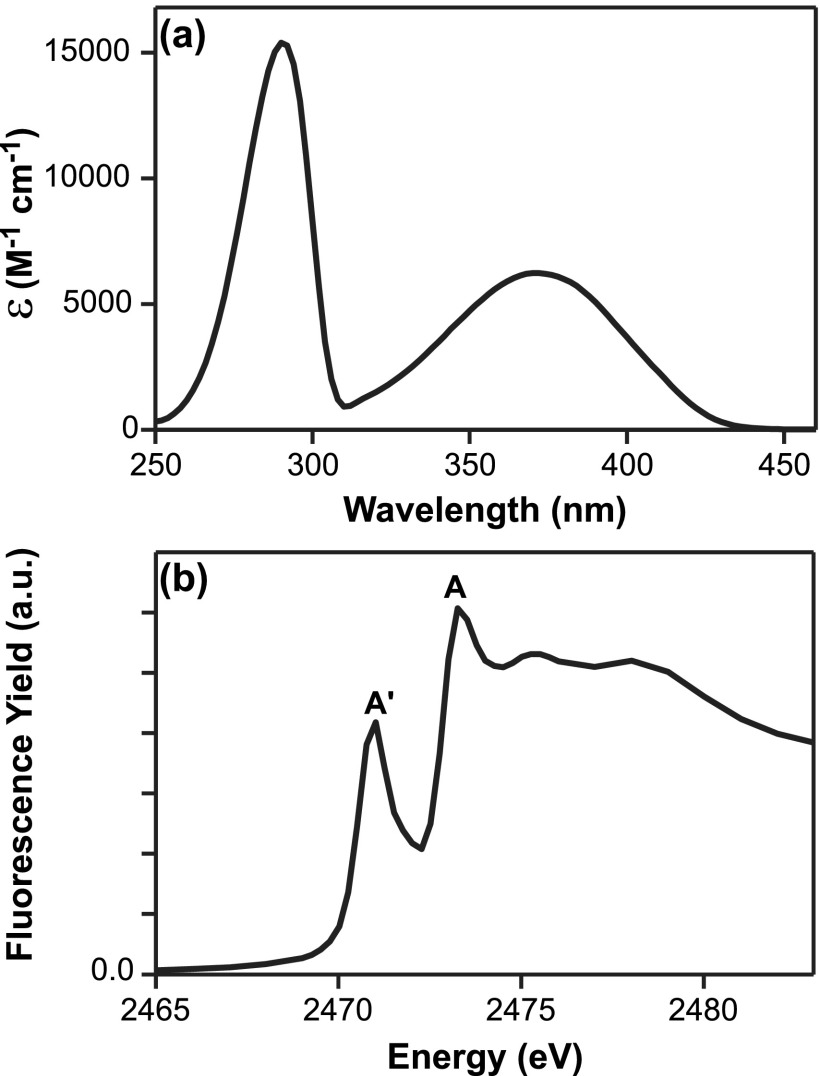
(a) UV/Visible spectrum of 2TP in acetonitrile absorptions at 370 nm and 280 nm. (b) Fluorescence yield-detected X-ray absorption spectrum of 2TP in acetonitrile. Two main near-edge features labeled A′ and A are observed at 2471.0 eV and 2473.4 eV.

The optical absorption spectrum of 2TP dissolved in acetonitrile is shown in Figure 2(a). The lower frequency absorption band is centered at 370 nm. This band is attributed to the excitation from the π-type HOMO, which has electron density on the S atom to the π*-type LUMO, S_2_. The higher energy absorption band is centered at 290 nm and is associated with a transition from the HOMO to the LUMO + 1 state also with the π* character. Previous studies have shown a solvent-dependent shift in the 2TP/2MP equilibrium and used the presence of the lower energy peak as evidence for the 2TP state.^40^ The existence of the band at 370 nm in Figure 2(a) provides evidence that 2TP is the predominant tautomer present in acetonitrile. Figure 2(b) shows the static S K-edge spectrum of 2TP in acetonitrile. There are two well-defined spectral features labeled A′ and A at 2471.0 eV and 2473.4 eV, respectively. Both features have been previously identified in the S K-edge spectra of merocyanine dye molecules that contain a terminal thione group.^53^ These single crystal polarized S K-edge measurements assigned the A′ feature to a S 1s → π* transition. The A feature polarized primarily along the C=S bond and assigned to an S 1s → σ* transition. The presence of the A′ feature is additional evidence that 2TP is the dominant tautomer in acetonitrile.

**FIG. 3.**
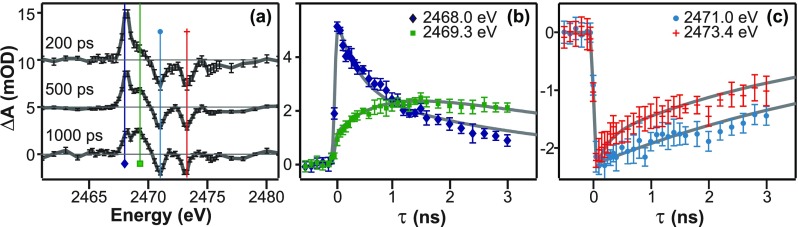
(a) Differential XA spectra at several delay times for 2TP pumped by 400 nm pulses. (b) Time-dependence of the XA spectrum at specific energies corresponding to the growth and decay of the transient product species and (c) the recovery of the ground state features of 2TP.

Figure 3(a) shows the differential pump-probe spectra at the S K-edge for several delay times (τ) between the 400 nm pump and X-ray probe. We identify four transient features in the difference spectra. Two negative bleaches are observed at 2471.0 eV and 2473.4 eV, which correspond to the energies of the A′ and A features identified in the equilibrium spectrum shown in Figure 2(b). The positive features at 2468.0 eV and 2469.3 eV in Figure 3(a) denote the presence of transient photochemical products formed in the solution. Figures 3(b) and 3(c) show the time-dependence of the transient absorption and bleach features, respectively. It is clear from these four traces that there are multiple time scales accompanying the excited state dynamics of 2TP dissolved in acetonitrile. All initial responses are limited by the 70 ± 10 ps duration of synchrotron based X-ray pulses except the 2469.3 eV spectral feature, which grows more slowly at 400 ps (Fig. 3(b)). The traces in Figures 3(b) and 3(c) show the longer time scale dynamics of 15 ns for the 2469.3 eV feature and 3–6 ns for the other spectral features.

The energies and time scales of the transient spectral features shown in Figure 3 are summarized in Table I. Growth and decay parameters are fit to a biexponential function convolved with an error function representing the X-ray pulse duration-limited instrument response. In cases where there was only one significant component, a single exponential fit was used. The bleach features at 2471.0 and 2473.4 eV represent ground state population depletion within the instrument response time with a long recovery time of a few nanoseconds. The two excited state absorption features exhibit distinct formation and decay dynamics. The 2468.0 eV transient absorption feature forms within the instrument response and then decays on a 0.4 ± 0.1 ps timescale followed by a longer 2.8 ± 0.8 ns decay component. On the other hand, the 2469.3 eV feature grows in on a 0.4 ± 0.1 ns timescale and then slowly decays on a 15 ± 4 ns timescale. The slow formation and the difference in the long-timescale recovery of the ground state indicate that the system returns to the 2TP ground state via multiple pathways, and the relatively slow growth of the 2469.3 eV feature reveals the presence of an intermediate photochemical transient.

**TABLE I.** Fitted parameters of biexponential decays of S K-edge XA features in time-resolved measurements of 2TP following photoexcitation. *No significant fast component detected from fitting.

Feature (eV)	A_fast_ (mOD)	τ_fast_ (ns)	A_slow_ (mOD)	τ_slow_ (ns)
2468.0	2.3 ± 0.7	0.4 ± 0.1	2.8 ± 0.7	2.8 ± 0.8
2469.3	−1.7 ± 0.2	0.4 ± 0.1	2.6 ± 0.2	15 ± 4
2471.0	*	*	−2.3 ± 0.1	5.6 ± 0.5
2473.4	−1.7 ± 0.1	0.1 ± 0.06	−1.7 ± 0.7	4.3 ± 0.5

**FIG. 4.**
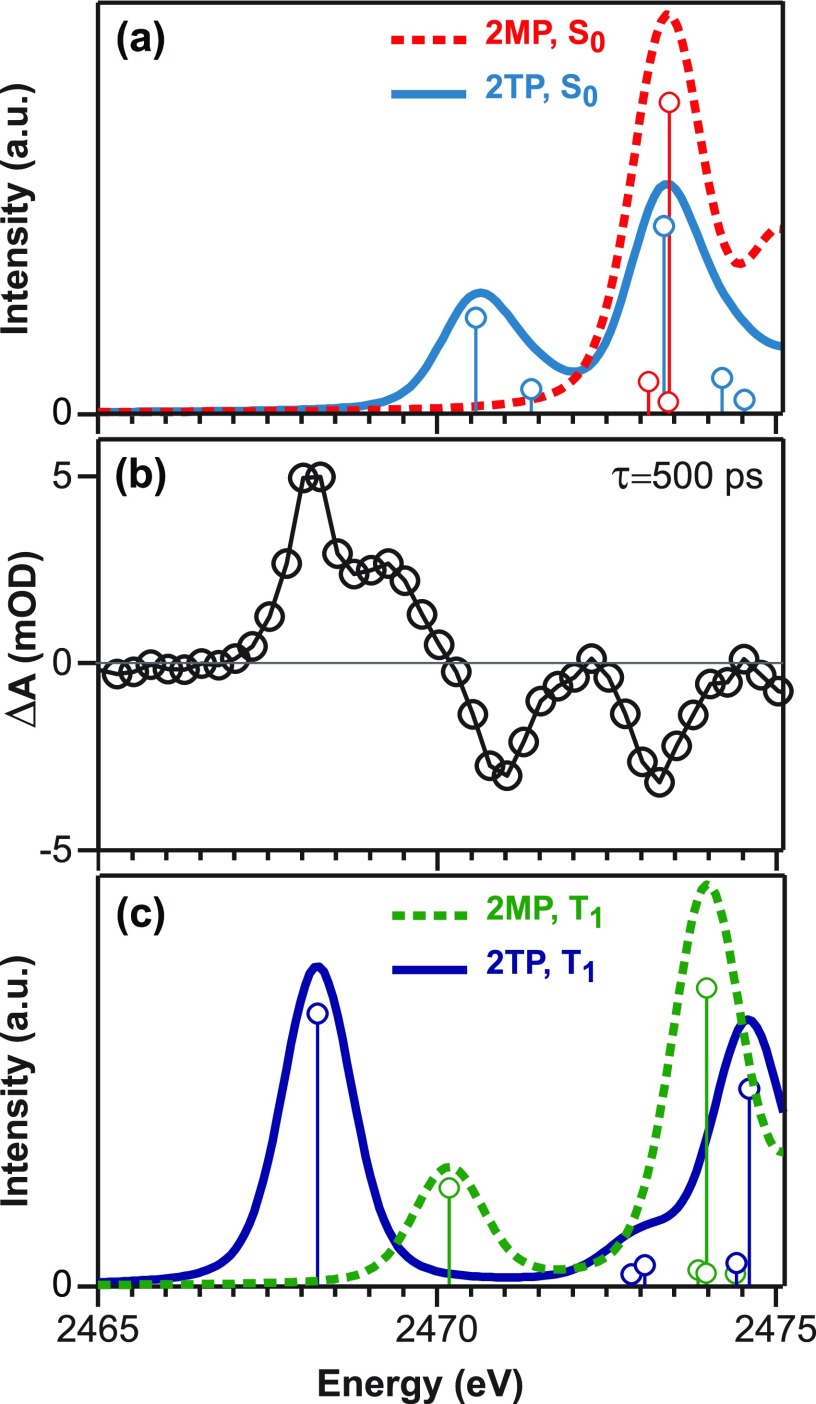
Comparison of the 500 ps time-resolved XA spectrum (b) with spectra calculated by TDDFT for the S K-edge of (a) singlet 2MP and 2TP and (c) triplet states of 2MP and 2TP.

In order to characterize the electronic structure of the ground electronic state and better interpret time-resolved signals, we performed TDDFT calculations, and the results are shown in Figure 4. The calculated XA spectra for the ground state of 2TP and 2MP are shown in Figure 4(a). The TDDFT calculations agree well with the observed experimental peak positions and amplitudes of near-edge transitions of 2TP at the S K-edge shown in Figure 2(b). The measured equilibrium XA features of 2TP at 2471.0 eV (A′) and 2473.4 eV (A) shown in Figure 2(b) agree with calculated ground state features of 2TP at 2470.6 eV and 2473.3 eV shown in Figure 4(a). The optimized density functional theory (DFT) structures reveal that the largest geometric perturbation near the S site following tautomerization is the elongation of the C-S bond by 0.03 Å between the triplet 2TP and 2MP states. We have included the coordinates of the optimized DFT structures in the supplementary material for interested readers. The TDDFT calculation generates difference density maps for each of the transitions contributing to the observed XA spectral features. The positive part of the difference densities maps the spatial extent of the core-excited electron and is plotted in Figure S1 in the supplementary material for representative transitions. The difference density maps show that the A′ feature is an S 1s → π* transition. The A feature is assigned to 1s → σ* symmetry with electron density along the C=S bond. The TDDFT calculations agree with previous assignments of the XA spectral features in thiones as described earlier.^53^ The calculated S K-edge XA spectrum of the 2MP ground state shows a single feature at 2473.4 eV. The positive part of the difference density for contributing transitions is plotted in Figure S2 (supplementary material) and reveals that the single feature has the S 1s → σ* character where the core-excited electron is distributed along the C-S and S-H bonds. The change in the C-S bond order and ring conjugation results in the absence of the A′ feature in the 2MP XA spectrum. Because the excited state absorption features observed in the transient XA spectra in Figure 4(b) appear at energies below that of the A′ feature in 2TP, these features cannot be explained solely by the formation of 2MP in its ground state. We note that it is possible that 2MP forms in its ground state in addition to other transient species identified by the X-ray measurement. This is because the TDDFT calculations suggest that the 2MP ground state would absorb at the same energy as the A bleach feature in the transient XA spectrum.

Previous studies have implicated the formation of triplet states in the photo-tautomerization of 2TP in polar solvents.^40,43^ The calculated S-K edge spectrum of the lowest-lying triplet state of 2TP is plotted in Figure 4(c), and the positive part of the difference densities for the transitions is shown in Figure S3 (supplementary material). There are two features in the calculated spectrum that are found at 2468.2 eV and 2474.6 eV. The lower energy feature is assigned to a core 1s → π* symmetry with electron density on the S atom and the C atoms on the ring. The higher energy feature is assigned to a 1s → σ* symmetry with electron density predominantly along the C=S bond. The low energy feature is in excellent agreement with the experimental excited state absorption at 2468.0 eV in Figure 4(b), suggesting the formation of the 2TP triplet upon photoexcitation. The calculated S-K edge spectrum of the lowest-lying triplet state of 2MP is also plotted in Figure 4(c), and the positive part of the difference densities for the transitions is shown in Figure S4 (supplementary material). The calculated spectrum of triplet 2MP shows two features at 2470.2 and 2474 eV. The higher energy feature is assigned to S 1s → σ* symmetry with electron density along the C-S and S-H bonds. We associate the 2470.2 eV feature in the calculated spectrum with the excited-state absorption feature at 2469.3 eV in the transient XA spectrum and propose the formation of the triplet 2MP species following photoexcitation. We considered numerous electronic states and conformers of 2TP/2MP to assign the identity of the 2469.3 eV feature in Fig. 4(b). In addition to the calculations in Figure 4, disulfide and deprotonated species were also considered (see Figure S5 (supplementary material)), but only 2MP in the triplet state contains a feature between the experimental energies of the 2468 eV transient and the A′ bleach. We also expect the energy of the 2470.2 eV feature in the calculation to be sensitive to solvent effects which are only treated at the continuum level here. Therefore, the 2MP triplet is the most plausible assignment for the 2469.3 eV transient.

**FIG. 5.**
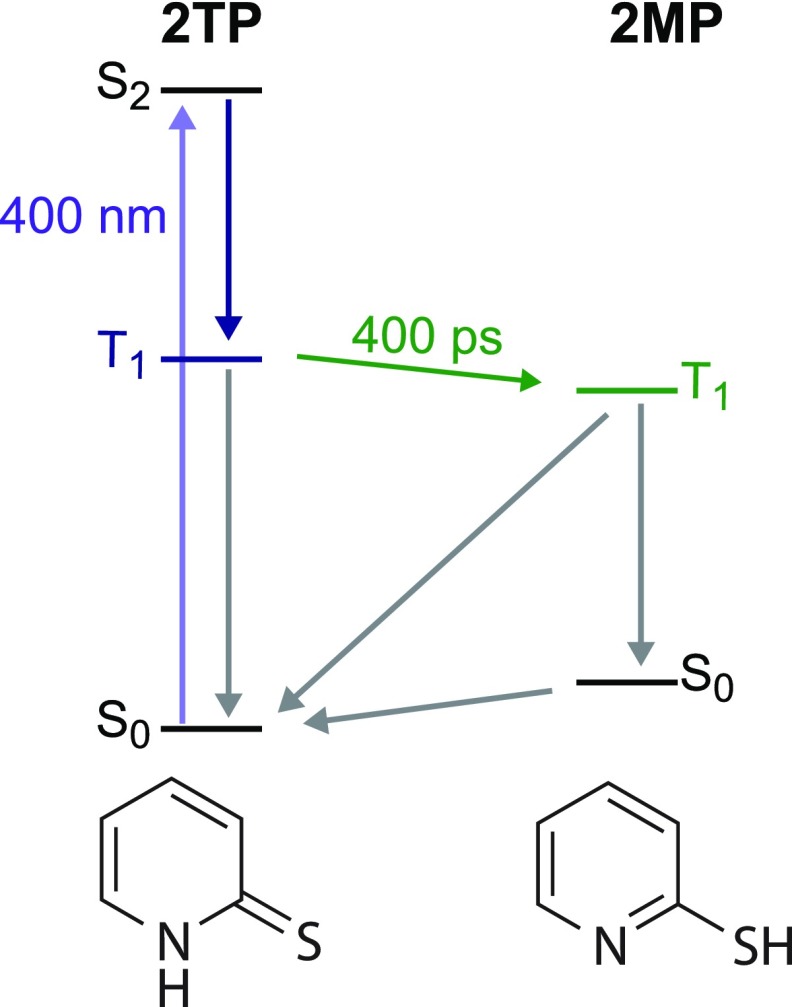
The proposed photochemistry of 2TP as viewed by time-resolved S K-edge XA spectroscopy. The 400 nm pulse populates the initial excited state, which undergoes a fast (<70 ps) intersystem crossing (ISC) to a triplet state. 2TP and 2MP triplet states show a 400 ps decay and rise, respectively. The gray arrows depict multiple possible pathways by which the triplet states could decay.

The data in Figures 3 and 4 and timescales listed in Table I suggest the photochemical pathway proposed in Figure 5. After excitation to the S_2_ state, 2TP undergoes a rapid intersystem crossing to its lowest lying triplet state of 2TP. This takes place within the instrument response time as indicated by the instantaneous appearance of the absorption feature at 2468.0 eV. The triplet 2TP state decays on two timescales. There is a 400 ps component and a 2.6 ns contribution. The fast component of the 2TP decay is in good agreement with the formation timescale of the 2MP triplet, suggesting that some fraction of the 2TP triplet is converted to a 2MP triplet. It is difficult to determine the concentration of triplet 2MP that is formed because we have no information about the extinction coefficients of the triplet states. However, normalizing the amplitudes of the fast and slow components for the 2TP triplet (2468.0 eV) decay suggests that 45% of the initially excited 2TP is converted to 2MP. The 2.6 ns component of the 2TP decay is likely associated with the return of the 2TP to the singlet ground state or the formation of additional undetected photoproducts. The triplet 2MP state decays on a much longer timescale of 15 ns. Figure 5 depicts two possible pathways that the 2MP triplet could return to the ground state. While our measurements clearly identify formation and decay timescales of the triplet states, we cannot resolve the precise pathways by which the system returns to the ground state or forms additional photoproducts.

We now compare our results to previous photochemical studies of 2TP in the solution. The prior resonance Raman study observed the formation of the ground state singlet 2MP on a timescale of 10 s of nanoseconds following 266 nm excitation of 2TP in water.^38^ While the relaxation lifetime of 15 ± 4 ns of the triplet 2MP state is consistent with their observed time scale for forming 2MP in the ground state, our calculations show that singlet ground state 2MP, triplet 2MP, and singlet ground state 2TP all have intense absorptions at the energy of the A feature. This makes it difficult to distinguish the signature of singlet 2MP from other possible transient species. It should also be noted that the Raman study was carried out in water and used 266 nm excitation. The different excitation wavelength could result in a different photochemical pathway for 2TP, and there could be significantly different reaction mechanisms in protic and aprotic solvents. This observation is supported by the recent femtosecond X-ray emission measurements at the N-Kedge which measured that aqueous 2TP is deprotonated within 150 fs following 400 nm excitation.^21^ It is also possible that the formation of 2MP may involve proton transfer to and from the solvent. To further investigate whether a deprotonated species could be identified using S K-edge measurements, the spectrum of the deprotonated triplet state was calculated (see Figure S5 in the supplementary material). We see that both the protonated and deprotonated molecules have a feature at 2468 eV. Consequently, while the S K-edge measurements are conclusive about the formation of a triplet state, they are not sensitive to the presence of a proton bound to the N site.

The important role of the solvent has previously been noted in flash photolysis studies.^40,43^ In Ar-saturated solutions, it was concluded that 2MP forms in protic solvents such as water, but in aprotic solvents such as acetonitrile, 2MP was not observed. In air-saturated solutions of acetonitrile 2MP was once again observed, and it was suggested that singlet oxygen plays a role in the reaction. It is possible that the acetonitrile solvent used in the experiment contained some amount of water since the solvent was not dried prior to experiments. This water could act as a proton donor and acceptor, and both the rate of 2TP formation and product yield would likely be dependent on the water concentration. We also note that in our measurements, no differences in the time resolved spectrum were observed when nitrogen was bubbled through the solvent prior to sample preparation. Finally, we note that previous quantum chemical calculations have determined a large reaction barrier (46.7 kcal/mol) between the 2TP triplet and 2MP triplet.^38^ While this is certainly a prohibitive barrier under equilibrium conditions, these calculations did not take into account explicit solvent interactions or the possibility that the solvent abstracts a proton from 2TP and donates one to 2MP. Moreover, it is well known that solvent rearrangement can greatly affect the barrier height for ESPT reactions.^23^ Future studies of the photochemistry of 2TP in other solvents will address these details.

## CONCLUSIONS

In summary, we demonstrate transient S K-edge XA spectroscopy of an organosulfur compound in solution on the hundreds of ps timescale. Our experiments show that S K-edge XA spectroscopy is a viable tool to probe proton transfer and other charge transfer processes in S containing molecular, biological, and material systems in the condensed phase. Using a combined experimental and TDDFT computational approach, we observe the formation of transient photochemical species following 400 nm excitation of 2TP in acetonitrile solution. We propose a photochemical reaction pathway where the triplet state of 2TP is formed within our instrument time resolution and the lowest triplet state of 2MP is formed on a 400 ps timescale. The ground state of the reactant species recovers on the nanosecond timescale. Femtosecond X-ray absorption spectroscopy at the N and S K-edges holds promise for detailing excited state proton transfer pathways following photoexcitation of 2TP.

## SUPPLEMENTARY MATERIAL

See supplementary material for the difference densities from the TDDFT calculations for various X-ray transitions, the effect of deprotonation in the 2TP triplet state, and the coordinates of the optimized geometries.
